# Comparative plastomes and phylogenetic analysis of seven Korean endemic *Saussurea* (Asteraceae)

**DOI:** 10.1186/s12870-022-03946-6

**Published:** 2022-11-29

**Authors:** Seona Yun, Seung-Chul Kim

**Affiliations:** 1 Department of Biological Sciences, Sungkyunkwan University, 2066 Seobu-ro, Suwon, Gyeonggi-do 16419 Korea; 2 Present Address: Department of Environmental Biology, State University of New York College of Environmental Science and Forestry, One Forestry Drive, Syracuse, NY 13210 USA

**Keywords:** Comparative analyses, Mitochondrial DNA, Mutation hotspots, Plastid genome, *Saussurea*

## Abstract

**Background:**

*Saussurea* is one of the most species-rich genera in the Cardueae, Asteraceae. There are approximately 40 *Saussurea* species distributed in Korea, with nearly 40% of them endemics. Infrageneric relationships remain uncertain due to insufficient resolutions and low statistical support. In this study, we sequenced the plastid genomes of five Korean endemic *Saussurea* (*S. albifolia, S. calcicola, S. diamantica, S. grandicapitula*, and *S. seoulensis*), and comparative analyses including two other endemics (*S. chabyoungsanica* and *S. polylepis*) were conducted.

**Results:**

The plastomes of Korean endemics were highly conserved in gene content, order, and numbers. Exceptionally, *S. diamantica* had mitochondrial DNA sequences including two tRNAs in SSC region. There were no significant differences of the type and numbers of SSRs among the seven Korean endemics except in *S. seoulensis.* Nine mutation hotspots with high nucleotide diversity value (*Pi* > 0.0033) were identified, and phylogenetic analysis suggested that those Korean endemic species most likely evolved several times from diverse lineages within the genus. Moreover, molecular dating estimated that the Korean endemic species diverged since the late Miocene.

**Conclusions:**

This study provides insight into understanding the plastome evolution and evolutionary relationships of highly complex species of *Saussurea* in Korean peninsula*.*

**Supplementary Information:**

The online version contains supplementary material available at 10.1186/s12870-022-03946-6.

## Background

*Saussurea* DC. (ca. 400 species) is one of the most abundant genera of the tribe Cardueae (Asteraceae) and is adapted to cool temperate and arctic regions of Asia, Europe, and North America [1, 2]. Lipschitz (1979) classified *Saussurea* into six subgenera according to morphological characteristics: *Amphilaena* (Stschegl.) Lipsch., *Eriocoryne* (DC.) Hook. f., *Frolovia* (DC.) Lipsch, *Jurinocera* (Baill.) Lipsch., *Saussurea* DC., and *Theodorea* (Cass.) Lipsch. However, several phylogenetic studies based on morphological traits and molecular markers have provided evidence to designate subg. *Jurinocera*, subg. *Frolovia*, subg. *Saussurea* sect. *Elatae,* subg. *Saussurea* sect. *Aucklandia,* and subg. *Saussurea* sect *Jacea* as new genera [3–6]. Subsequently, *Saussurea* has recently been recognized as four subgenera (*Amphilaena*, *Eriocoryne, Saussurea,* and *Theodorea*) [6]. Although several phylogenetic studies have been conducted using nuclear and plastid loci as molecular markers [3–5, 7], relationships within *Saussurea* were poorly resolved due to rapid adaptive radiation and convergent evolution [5, 7, 8].

Recently, the advent of next generation sequencing technologies has led to rapidly accumulation of genomic data. Because of conserved structure, non-recombinant traits, and greater variability than the mitochondrion genome, plastid genome (plastome) regions have consistently been used as a robust tool in phylogenetic studies [9–11]. Furthermore, studies using complete plastomes have offered new insights into phylogenetic relationships and the diversification histories of species [12–14]. Although studies on phylogenetic relationships, origins, and evolution using plastomes of *Saussurea* have been reported [15, 16], the limited number of Korean species was used in the previous studies, and there is a need for a better understanding the relationship among Korean species.

*Saussurea* is one of the rich species groups in the flora of Korea. The approximately 40 *Saussurea* species distributed in Korea comprise 2 subgenera, *Theodorea* and *Saussurea*, and nearly 40% are endemic species belonging to subg. *Saussurea*: *S. calcicola* Nakai, *S. chabyoungsanica* Im, *S. chinnampoensis* H. Lév. & Vaniot, *S. conandrifolia* Nakai, *S. diamantica* Nakai, *S. eriophylla* Nakai, *S. grandicapitula* W. Lee et H. T. Im, *S. koidzumiana* Kitam., *S. macrolepis* (Nakai) Kitam., *S. myokoensis* Kitam., *S. polylepis* Nakai, *S. rorinsanensis* Nakai, *S. seoulensis* Nakai, and *S. uchiyamana* Nakai [17]. Obtaining a rigorous phylogenetic framework for *Saussurea* species in Korea has been exceptionally challenging due to sampling difficulties, insufficient levels of resolutions, and degree of statistical support. In particular, the phylogenetic tree based on chloroplast (cp) DNA markers (e.g., *trn*L–*trn*F and *trn*H–*psb*A) commonly used as barcodes has not yet been clearly resolved, showing multiple polytomies (Yun and Kim, unpublished data).

In this study, plastomes of five Korean endemic species, *S. albifolia, S. calcicola, S. diamantica*, *S. grandicapitula,* and *S. seoulensis,* were sequenced and comparative analyses were conducted, including two previously reported species *S. chabyoungsanica* [18] and *S. polylepis* [19]. *Saussurea albifolia,* described recently as a new species, has cordate or deltoid-cordate leaves with white or yellowish hair on the abaxial surface. In addition, the campanulate involucre has brown-cobwebby hair and the tips of phyllaries do not recurve [20]. *Saussurea calcicola* has large leaves with cobwebby hair on the underside, and wings on the petiole. It grows to approximately a height of 1 m in limestone regions. *Saussurea grandicapitula* has cobwebby hairs on the petioles of the radical and lower cauline leaves, big globose involucres with brown-cobwebby hairs, and recurved phyllaries [21]. *Saussurea albifolia*, *S. diamantica,* and *S. seoulensis* share common traits including basal rosette leaves, cobwebby hair on abaxial leaf surfaces, and white or yellowish hairs on the involucre. However, *S. diamantica* has recurved involucres, and *S. albifolia* has larger involucral width than *S. diamantica* and yellowish cobwebby hair on the abaxial surface of leaf. *Saussurea seoulensis* has a distinctive bell-shaped involucre, the largest such structure among the species [17]. The most notable differences between *S. chabyoungsanica* and other Korean *Saussurea* species are long lanceolate leaves with short petioles and a compact corymb [22]. *Saussurea polylepis* is distinguishable by its glossy and reniform leaves. Based on several diagnostic morphological features of *Saussurea*, congeneric species are distinguished, but variable molecular markers through comparison of plastomes of endemic species can overcome the low resolution shown in the previous phylogenetic study (Yun and Kim, unpublished data). In addition, identifying the structure and characteristics of plastomes of the Korean endemic *Saussurea* species will provide insights into understanding the plastome evolution of *Saussurea*.

The aims of this study were (1) to determine five complete plastomes of Korean endemic species, (2) to identify divergent sequence hotspots for the development of informative cpDNA markers, (3) to gain insight into the evolution of *Saussurea* plastomes, including structural differences and molecular evolutionary patterns, and (4) to reconstruct phylogenetic relationships among the Korean endemic *Saussurea* species and estimate their divergence times using plastomes.

## Results

### Characteristics of plastomes

**Fig. 1:**
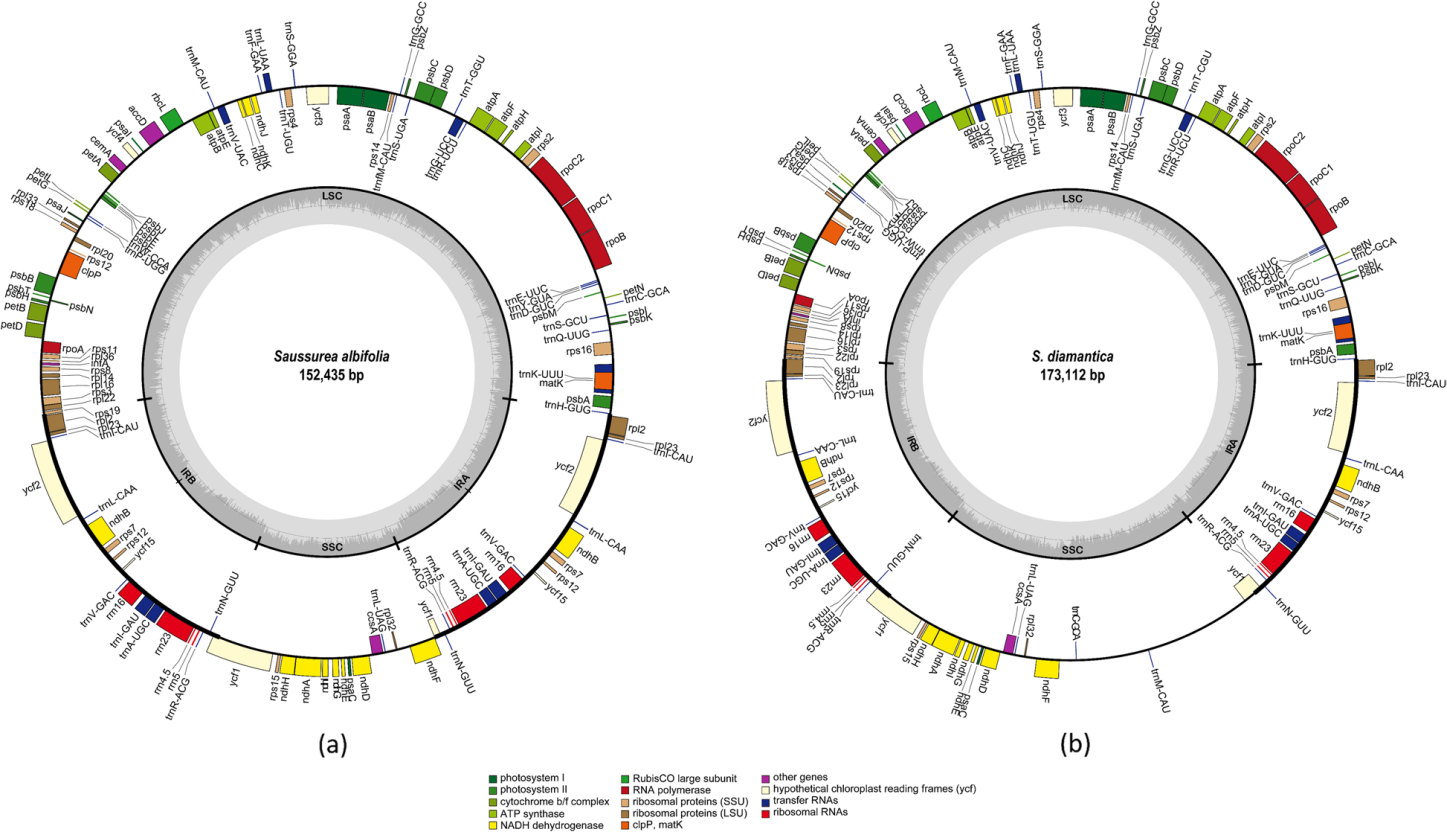
Gene maps of *Saussurea* plastid genome. (**a**) *S. albifolia.* (**b**) *S. diamantica.* The genes inside and outside the circle are transcribed in the clockwise and counterclockwise directions, respectively. Genes belonging to different functional groups are shown in different colors. The gray area in the inner circle indicates guanin–cytosine (GC) content while the lighter gray area shows adenosine–thymine (AT) content. *S. calcicola, S. grandicapitula,* and *S. seoulensis* share the same plastome structure in terms of gene contents and gene order with *S. albifolia* despite their different length

Newly sequenced plastomes of the five species had a total length of 152,435 (*S. albifolia*) – 173,114 bp (*S. diamantica*) (Fig. 1 and Table 1). Because some parts of mitochondrial DNA sequences including two tRNAs were inserted in the small single copy (SSC) region (*ndh*F-pseudo *ycf*1) of plastome of *S. diamantica*, the total length of *S. diamantica* was longer than that of other *Saussurea* species by 20,550 bp (Fig. 1b and Table 1). BLAST searches were performed to determine the characteristics of the insertion. The result demonstrated that the inserted sequences were highly matched to *Chrysanthemum, Diplostephium, Lactuca, Helianthus,* and *Paraprenanthes* mitochondrial DNA sequences, ranging from 5,159 bp (*Paraprenanthes diversifolia,* MN661146) to 7,090 bp (*Diplostephium hartwegii,* KX063855), but this does not mean the continuous consistency of the whole 20,550 bp on the mitochondrial genome. The transfer of mitochondrial DNA sequences to plastoms has been reported in the families Apiaceae, Apocynaceae, and Poaceae [23–25]. The previous studies indicated that genes of less than 3 kb of mitochondrial DNA are inserted into the IR or LSC regions. Given that a plastome is highly conserved, the large insertion of mitochondrial DNA sequences is an unusual event. Thus, confirmation is needed that the insertion was not merely a product of assembling error. By comparing sequencing depth before and after the insertion, Ma et al. [25] confirmed that it is not a product of misassembly. Because the plastome occupies the smallest portion of the genomic DNA, it can be easily distinguished from mitochondrial and nuclear DNA sequences by sequencing depth. Ma et al. [25] also inferred that the nuclear and mitochondrial genomes are larger than the plastome and would have a lower sequencing depth. The average sequencing depth of *S. diamantica* was 322.3 and that of the inserted regions was 356.8. The average sequencing depth of the surrounding regions, which was 349.1 and 346.9 before and after the insertion with 200 bp, is similar to that of the inserted region. These results indicated that misassembly is not a cause of mitochondrial DNA insertion into the plastome of *S. diamantica*. In addition, PCR amplification and Sanger sequencing were conducted to confirm the insertion using designed two primer sets (SD1f: GTAGGGGGTGGGCGTATTTC, SD1r: GATGTCGAGTGCCGCTTTTC, and SD2f: AGGGTGATGCTTGGCTTCT, SD2r: TTTTCGTGGTTAGAGCGGCT), and amplifications were successful but not for other species (data not shown). It also supported that there was no error in the assembly process.

**Table 1:** Summary of seven *Saussurea* plastid genomes

	*S. albifolia*	*S. calcicola*	*S. chabyoungsanica*	*S. diamantica*	*S. grandicapitula*	*S. polylepis*	*S. seoulensis*
Reference	This study	This study	Cheon et al. [18]	This study	This study	Yun et al. [19]	This study
Total length (bp)	152,435	152,453	152,446	173,114	152,484	152,488	152,578
LSC length (bp)	83,387	83,399	83,397	83,482	83,431	83,417	83,441
SSC length (bp)	18,678	18,672	18,679	37,846	18,671	18,689	18,727
IRa length (bp)	25,185	25,191	25,185	25,893	25,191	25,191	25,205
Total GC content (%)	37.7	37.7	37.7	38.6	37.7	37.7	37.7
GC content of LSC	35.8	35.8	35.8	35.8	35.8	35.8	35.8
GC content of SSC	31.4	31.4	31.4	39.1	31.4	31.4	31.4
GC content of IR	43.1	43.1	43.1	42.8	43.1	43.1	43.1
Protein-coding gene number	80	80	80	80	80	80	80
rRNA gene number	4	4	4	4	4	4	4
tRNA gene number	30	30	30	32^a^	30	30	30
Total gene number	114	114	114	116	114	114	114

^a^indicates that *S. diamantica* additionally has two mitochondrial tRNAs (*trn*C-GCA and *trn*M-CAU) in SSC

Seven *Saussurea* species have a typical quadripartite structure comprising a pair of inverted repeats (IR: IRA and IRB) of 25,185–25,893 bp, separated by SSC of 18,671–37,846 bp and large single copy (LSC) of 83,387–83,482 bp. Other than *S. diamantica*, the length and GC content of IR, SSC, and LSC and gene content were the same (Table 1). The seven plastomes contained 114 identical genes, including 80 protein-coding genes, 30 tRNA, and four rRNA genes. Eighteen genes including 12 protein–coding genes (*atp*F, *clp*P, *ndh*A, *ndh*B, *pet*B, *pet*D, *rpl*2, *rpl*16, *rpo*C1, *rps*12, *rps*16, and *ycf*3) and 6 tRNA genes (*trn*A–UGC, *trn*G–UCC, *trn*I–GAU, *trn*K–UUU, *trn*L–UAA, and *trn*V–UAC) had intron, and 17 genes (*ndh*B, *rrn*4.5, *rrn*5, *rrn*16, *rrn*23, *trn*A–UGC, *trn*I–CAU, *trn*I–GAU, *trn*L–CAA, *trn*N–GUU, *trn*R–ACG, *trn*V–GAC, *rpl*2, *rpl*23, *rps*7, *ycf*2, and *ycf*15) were duplicated in IR regions (Table S1). However, *S. diamantica* had two additional tRNA genes (*trn*C–GCA and *trn*M–CAU) from the mitochondrial genome in the SSC region. The formation of tertiary structure of two tRNAs was confirmed by simulation through the tRNAscan-SE 2.0.

Seven *Saussurea* plastomes possessed the two inversions in LSC region like other Asteraceae and depicted high similarity at the LSC, IR, and SSC boundaries (Fig. S1). *Rps*19 was across the LSC–IRB boundary without any change in sequence length, and *trn*H–GUG was located three base pairs away from the LSC–IRA boundary in all species. *Ycf*1 gene crossed SSC–IRB, with 4,022–4,740 bp within the SSC region and 561–1,240 bp within the IRB region. *Ycf*1 was a pseudogene, located at the SSC–IRA boundary, with 6–752 bp within the SSC region and with 561–1,240 bp within the IRA. In particular, *S. diamantica* had longer *ycf*1 (pseudogene) than others.

### Identification of variable regions

SNP (single nucleotide polymorphism) patterns that can be divided into 2 groups were found in 42 regions (Table S2). Of them, 34 regions were in LSC, followed by SSC and IR. Based on *S. involucrata* as a reference, there were no large differences among the seven Korean endemics. The LSC and SSC regions were more divergent than IR regions, and the coding regions were more conserved than the non-coding regions (Fig. S2). However, coding regions such as *rbc*L in the LSC region, *ycf*1 in the SSC region, and *ycf*2 in the IR regions showed variability.

**Fig. 2:**
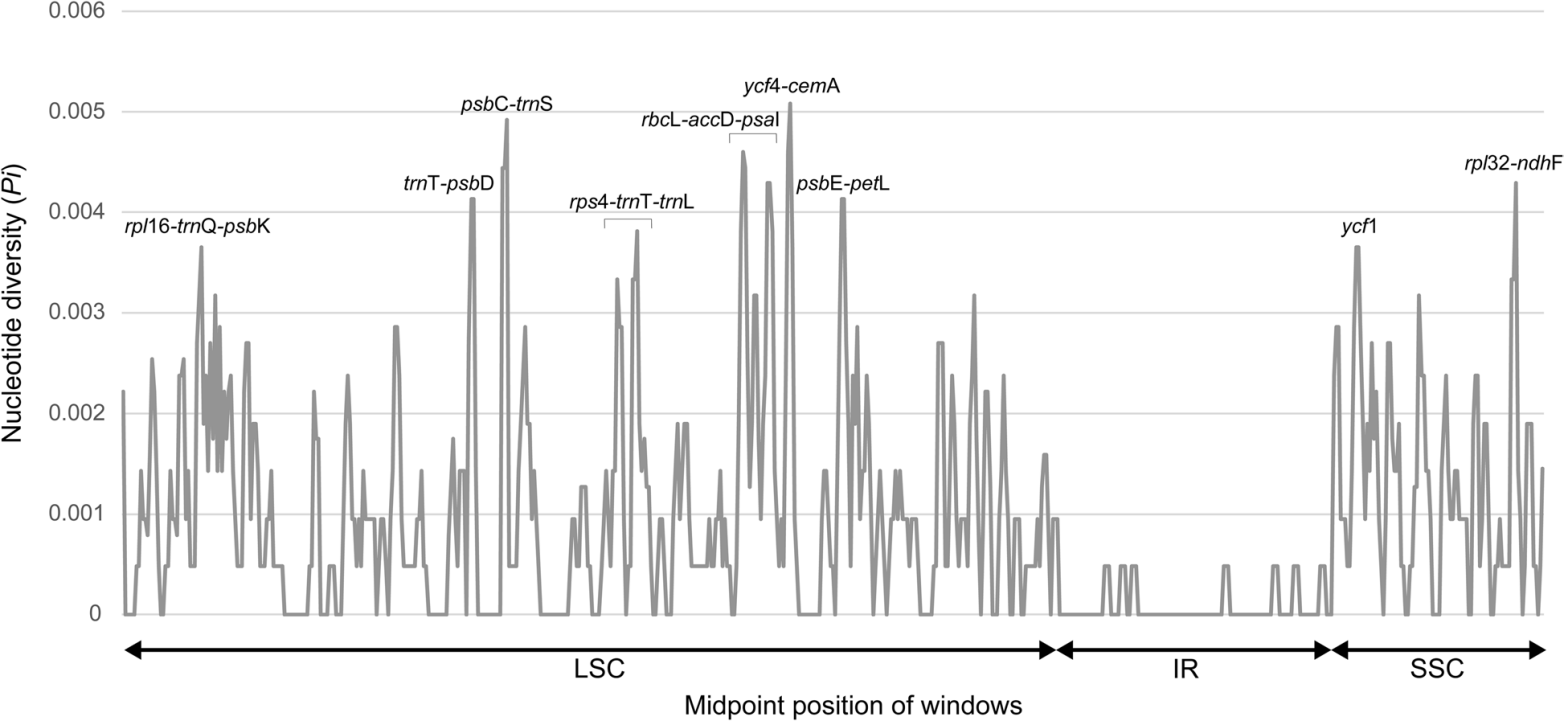
Nucleotide diversity graphs of the complete plastid genomes of seven *Saussurea*. The *x*-axis and *y*-axis respectively indicate midpoint position of each window and nucleotide diversity (*Pi*)

The nucleotide diversity (*Pi*) ranged from 0 to 0.0053 (Fig. 2). The IR region had a relatively low nucleotide diversity value, ranging from 0 to 0.00286. We detected nine divergence hotspots with *Pi* values over 0.0033. Among them, seven were located in the LSC region, and two were located in the SSC region. Other than *ycf*1, the variable regions were concentrated in intergenic spaces. The hotspot with the highest *Pi* was *ycf*4–*cem*A (0.0051), followed by seven intergenic regions (*psb*C–*trn*S, *rbc*L–*acc*D–*psa*I, *rpl*32–*ndh*F, *trn*T–*trn*D, *psb*E–*pet*L, *rps*4–*trn*T–*trn*L, and *rpl*16–*trn*Q–*psb*K) and one gene region (*ycf*1).

### Simple sequence repeats (SSRs) analysis

**Fig. 3:**
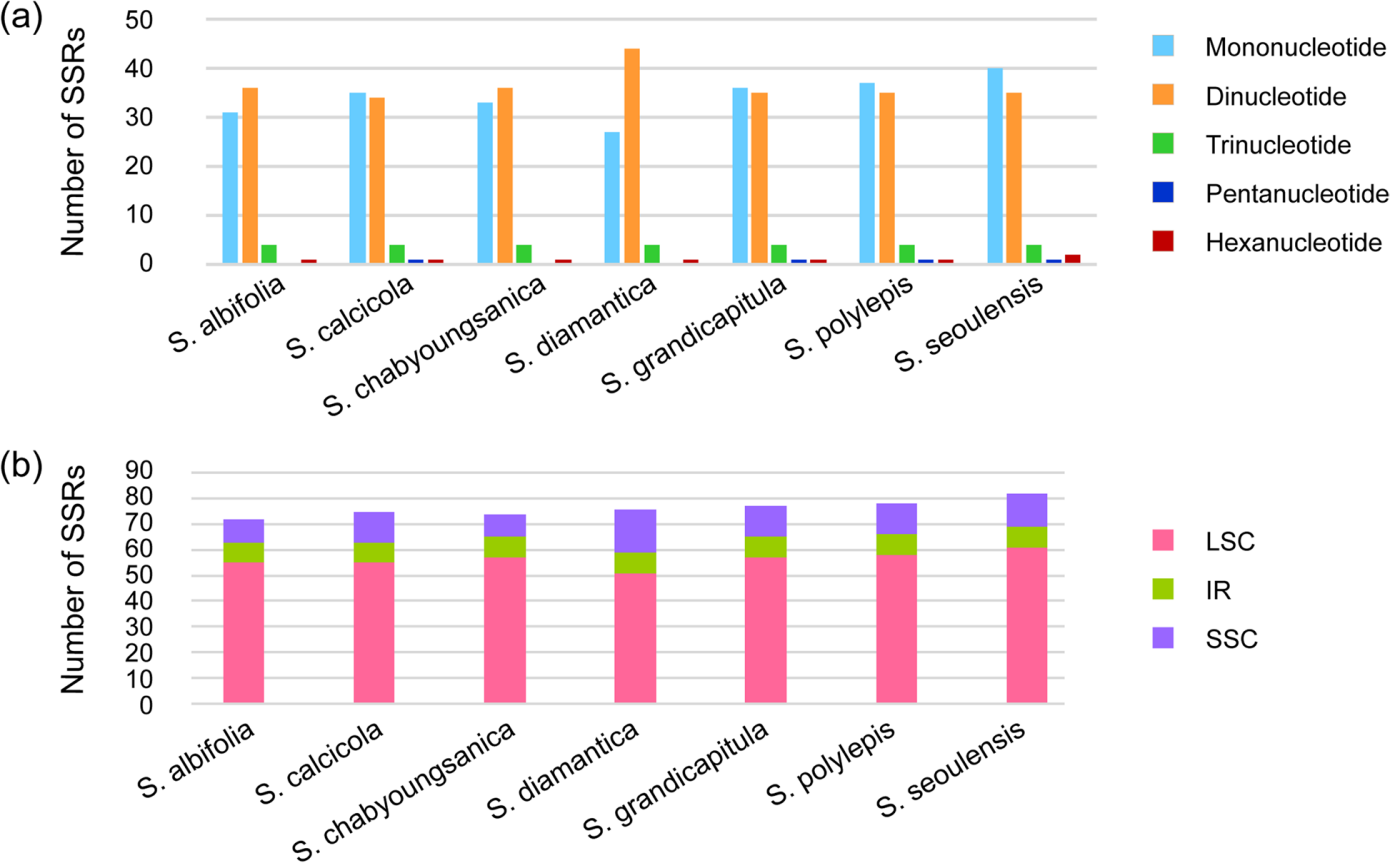
Information on simple sequence repeats on seven *Saussurea* plastid genomes. (**a**) SSR repeat types and frequencies; (**b**) Frequencies of SSRs in LSC, SSC, and IR regions

Five categories (mononucleotide, dinucleotide, trinucleotide, pentanucleotide, and hexanucleotide) of SSRs were detected, and the types and numbers of SSRs were similar across the seven *Saussurea* (Fig. 3). The total number of SSRs was 72 in *S. albifolia*, 75 in *S. calcicola*, 74 in *S. chabyoungsanica*, 76 in *S. diamantica*, 77 in *S. grandicapitula*, 78 in *S. polylepis*, and 82 in *S. seoulensis*. The detected SSRs were mainly located in the LSC region (67.1%–77%) and distributed in the IR and SSC regions ranging from 9.8% to 11.1% and from 12.2% to 22.4%, respectively. Twenty-three of the SSRs detected from the seven *Saussurea* were located in 15 genes (*cem*A, *ndh*B, *pet*A, *psa*A, *psb*C, *rbc*L, *rpo*A, *rpo*B, *rpo*C1, *rpo*C2, *rps*15, *rrn*23, *trn*S–UGA, *ycf*1, and *ycf*2) with 3–10 repeat numbers (Table S3). The most abundant type was mononucleotides A/T and species–specific SSR was identified from *S. seoulensis* as hexanucleotides TACAAA/TTTGTA.

### Synonymous and non-synonymous substitution rate analysis

The non-synonymous (Ka) to synonymous (Ks) substitution rate ratio (Ka/Ks) has been used to determine whether protein-coding genes are subjected to selective pressure. If Ka/Ks is greater than 1, it could indicate that it is under positive selection [26]. We calculated synonymous and nonsynonymous substitution rates between *S. involucrata* and Korean endemic *Saussurea* (Fig. S3). Approximately 90% of protein coding genes were below 1 in Ka/Ks values. In seven Korean species, the Ka/Ks value was close to zero at 12 protein coding genes (*clp*P, *ndh*B, *ndh*H, *pet*D, *psa*C, *psb*A, *psb*B, *psb*C, *psb*D, *rpo*B, *rps*11, and *rps*15) while that of five protein coding genes (*ndh*I, *psa*J, *psb*L, *rpl*33, and *ycf*2) were 50, indicating positive selection influenced the differentiation of *Saussurea*.

### Codon usage analysis

**Fig. 4:**
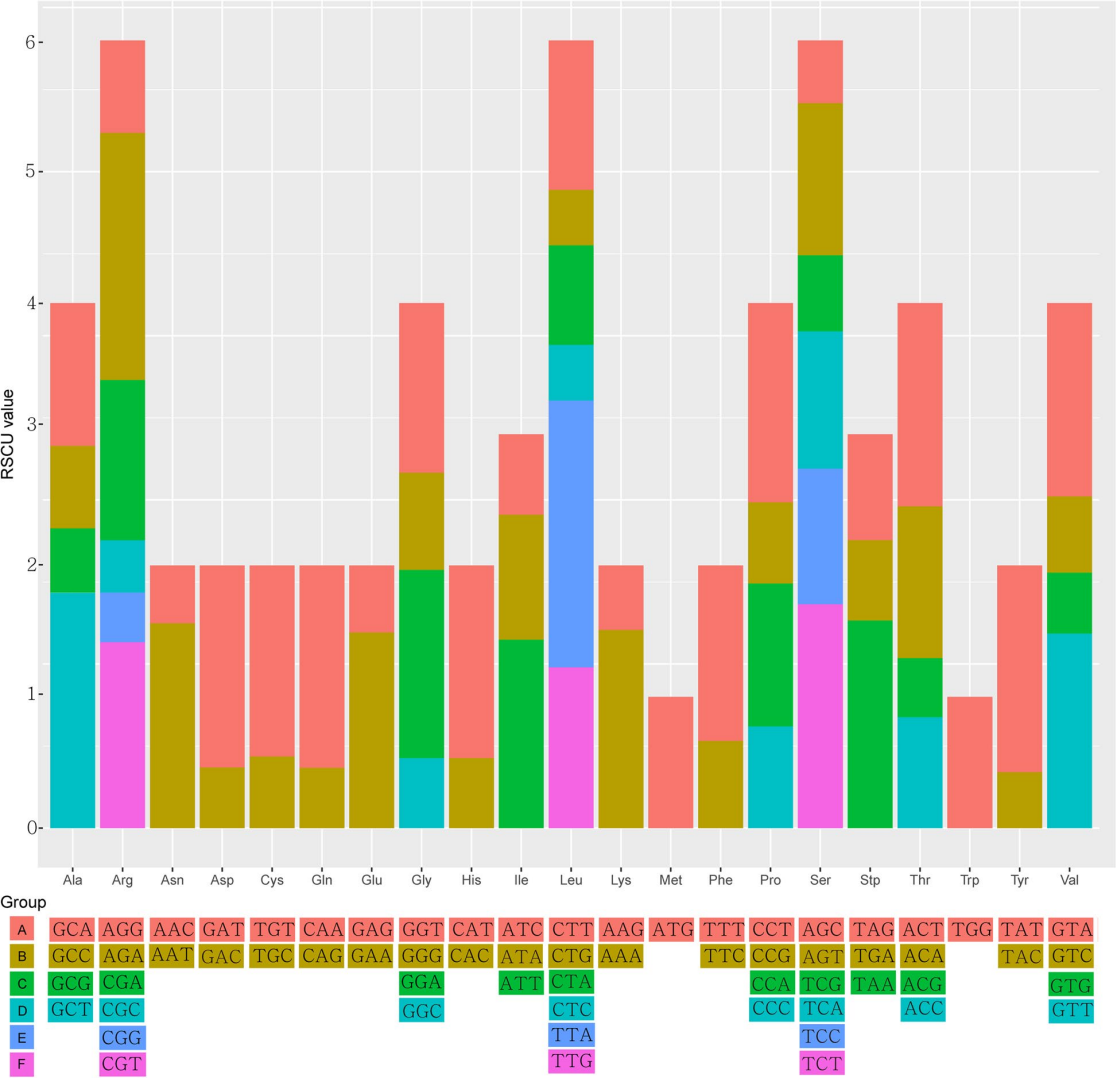
Codon content of 20 amino acids and stop codons in all protein-coding genes of the seven *Saussurea* plastid genomes

We detected similar patterns in the frequency of codon usage of seven Korean endemics. The 80 annotated protein-coding genes are encoded by 22,739 codons in *S. albifolia*, 22,831 in *S. calcicola* and *S. polylepis*, 22,826 in *S. chabyoungsanica,* 22,821 in *S. diamantica*, and 22,835 in *S. grandicapitula*, and 22,834 in *S. seoulensis* (Table S4). Leucine was the most abundant amino acid (10.6%), whereas cysteine was the least (1.1%). The most used synonymous codon was ATT, encoding isoleucine, and the least used was TGC, encoding cysteine. Usage of the start codon methionine (ATG) and tryptophan (TGG) had no biases (relative synonymous codon usage, RSCU = 1). All preferred relative synonymous codons (RSCU > 1) ended with an A or a T, other than TTG (leucine) (Fig. 4). The tendency for codon preference was similar among species. Of 61 codons (except for stop codon), 14 (Ala–GCT, Arg–AGA, Asn–AAT, Asp–GAT, Gln–CAA, Gly–GGA, His–CAT, Leu–TTA, Lys–AAA, Pro–CCT, Ser–TCT, Thr–ACT, Tyr–TAT, and Val–GTA) were highly preferred (RSCU > 1.5).

### Phylogenetic analysis and molecular age estimation

**Fig. 5:**
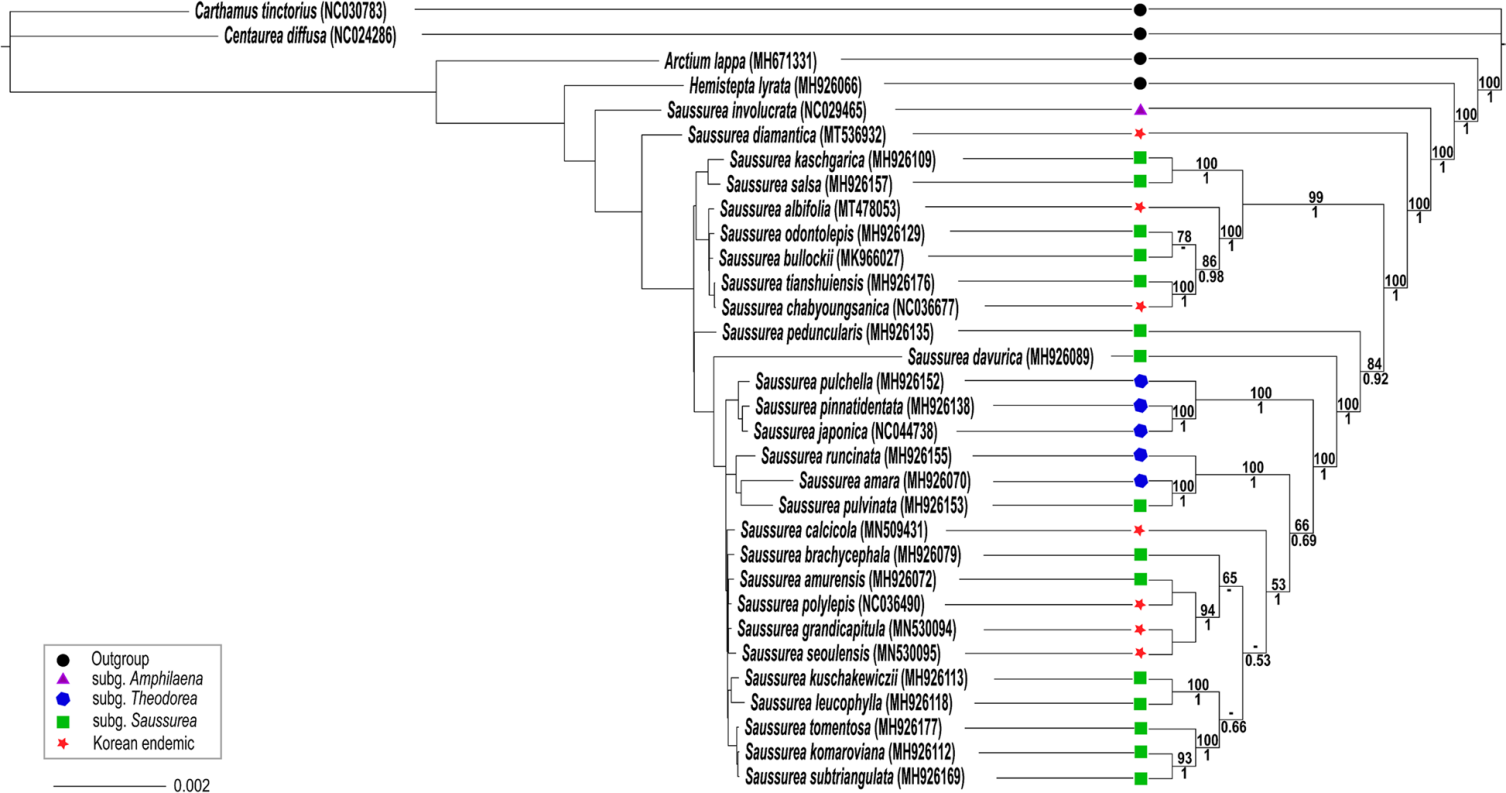
Maximum likelihood tree based on plastid genome sequences from 32 species of Cardueae. Bootstrap support values > 50% and posterior probability > 0.5 are shown at the branches

In this study, 32 plastomes were used to determine the phylogenetic relationships among Korean endemic *Saussurea.* As a result, the higher resolution phylogenetic tree showed that *Saussurea* based on the current sampling is not monophyletic (Fig. 5). Of the seven endemic species, *S. diamantica* diverged first. The morphologically similar *S. albifolia* and *S. seoulensis* did not form a sister relationship; *S. albifolia* formed a sister with the group including *S. odontolepis, S. bullockii, S. tianshuiensis,* and *S. chabyoungsanica*. Limestone endemic, *S. calcicola*, shared its common ancestor with the group consisting of *S. brachycephala, S. amurensis*, *S. polylepis, S. grandicapitula, S. seoulensis. S. kuschakewiczii, S. leucophylla, S. tomentosa, S. komaroviana,* and *S. subtriangulata.* However, relatively low bootstrap values hindered us to determine precisely their phylogenetic relationships. Also, *S. chabyoungsanica*, which is narrow limestone endemic to central Korea, is sister to *S. tianshuiensis*, which occurs narrowly in high montane meadows (1800–2500 m) in three provinces of northwestern China (i.e., SE Gansu, Shaanxi, and Ningxia).

**Fig. 6:**
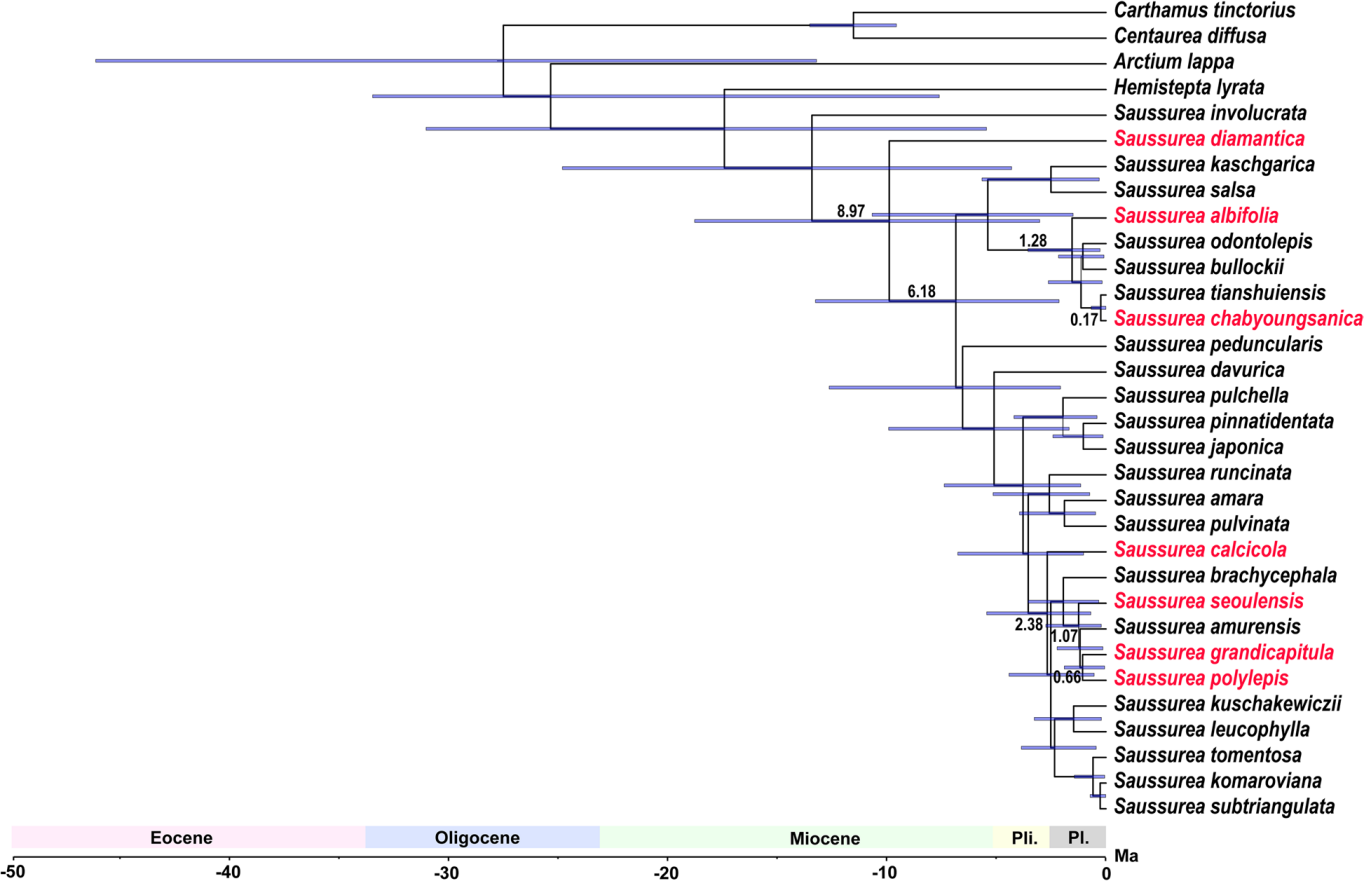
Divergence time estimates of Korean endemic *Saussurea* based on complete plastid genomes. Pale purple bars show 95% HPD credibility intervals. The numbers above or below branches represent median divergence time estimates. Pl. and Pli. indicate Pleistocene and Pliocene, respectively. Korean endemic species were marked in red

The molecular age estimation suggested that endemic Korean *Saussurea* originated in the late Miocene (Tortonian), with the estimated crown age of approximately 9 million years ago (95% HPD, 3.03–18.8 million years ago, MYA) (Fig. 6). The clade containing all but *S. diamantica*, which has unusual mitochondrial DNA sequences insertion, was estimated to be 6.18 MYA (95% HPD, 2.14–13.28 MYA). Two major lineages of the Korean endemics, i.e., *S. albifolia* – *S. chabyoungsanica* and *S. calcicola* – *S. seoulensis* – *S. polylepis* – *S. grandicapitula*, appear to be speciated even more recently, during the Pleistocene.

## Discussion

### Characterization of the Korean endemic plastid genome

Accumulation of plastome data from various land plants has improved our understanding of plant evolution. In general, the plastome has a highly conserved structure; a single circular DNA molecule is composed of a large single copy, a small single copy, and two copies of inverted repeats. However, structural rearrangements, gene loss, IR expansion and contraction, inversion, and gene transfer occur in certain species or lineages [27]. Like other angiosperms, Korean endemics have a quadripartite structure and are highly conserved in gene order, gene content, and gene number. As the extension and contraction of the IR region is a common phenomenon in angiosperms [28, 29], these changes are also found in Korean endemics and have affected the length of the plastome [30, 31].

Interestingly, we found that *S. diamantica* has mitochondrial DNA sequences including two tRNAs in SSC region. DNA transfer between nuclear and organellar (plastid and mitochondria) genomes has been reported in several taxa. Most prominent transfer is from organellar genomes into the nuclear genomes [32, 33]. Also, there are previous studies reporting gene transfer between organelle genomes [23, 25, 34]. In particular, the transfer of mitochondrial DNA sequences to plastomes has been reported in the families Apiaceae, Apocynaceae, and Poaceae [23–25]. However, the evidence of DNA transfer between organellar genomes has not been reported in Asteraceae. In this study, our result of BLAST search demonstrated that the inserted sequences were highly matched to *Chrysanthemum, Diplostephium, Lactuca, Helianthus,* and *Paraprenanthes* mitochondrial DNA sequences, but this does not mean the continuous consistency of the whole 20,550bp on the mitochondrial genome. Even though it is difficult to reveal whether the exact mechanism is the result of deletion after insertion of long DNA sequences or the result of multiple transfers. The insertion of mitochondrial DNA into the plastome is not common at the species level, so these conclusions require verification through the further studies. Information on the mitochondrial genome in *Saussurea* will improve understanding of the insertion event and genome evolution overall.

### Nucleotide diversity and selection pressure

The *Pi* values can provide useful information for marker development for phylogenetic analysis and DNA barcoding. The effectiveness of newly developed markers based on nucleotide diversity values has been verified through the discrimination of closely related species [35, 36]. In seven Korean species, *Pi* ranged from 0 to 0.0053 (Fig. 2), indicating high similarity of sequences among seven *Saussurea* species. These low values were reported in *Sonchus* (Asteraceae) ranged from 0 to 0.006 [37] and *Meconopsis* (Papaveraceae) ranged from 0 to 0.007 [38]. The high similarity might be related to the recent speciation.

The hotspot with the highest *Pi* was *ycf*4–*cem*A (0.0051), followed by seven intergenic regions (*psb*C–*trn*S, *rbc*L–*acc*D–*psa*I, *rpl*32–*ndh*F, *trn*T–*trn*D, *psb*E–*pet*L, *rps*4–*trn*T–*trn*L, and *rpl*16–*trn*Q–*psb*K) and one gene region (*ycf*1). Of the nine variable regions detected in this study, *rpl*16–*trn*Q, *trn*T–*psb*D, *rps*4–*trn*T–*trn*L, *acc*D–*psa*I, *psb*E–*pet*L, and *rpl*32–*ndh*F coincide with the variable cp regions identified by Shaw et al. [10, 11] and *trn*T–*psb*D, *rps*4–*trn*T–*trn*L, *acc*D–*psa*I, *psb*E–*pet*L, and *rpl*32–*ndh*F regions have been utilized in conducting phylogenetic studies in many taxa [39–43]. Previous studies used in cp molecular markers, such as *trn*L–*trnF*, *ps*bA–*trn*H, and *mat*K, have been poorly resolved in *Saussurea* [3–5]. The low resolution of phylogenetic studies can be interpreted as the low diversity values of *trn*L*-trn*F*, psb*A*-trn*H, and *mat*K markers. Therefore, using these identified variable regions will be helpful for further clarifying phylogenetic relationships.

As microsatellites or SSR markers have hyper-mutation rates and polymorphism, they are suitable for population genetic analyses such as population genetic structures and gene flow patterns. In particular, Powell et al. [44] suggested that the SSR markers from the chloroplast are useful for acquiring insight into gene flow related to seed and pollen dispersal, genetic structure, nuclear-chloroplast interaction, and the origin of polyploidy. Many studies have reported the use of cpSSR markers with high polymorphisms. For example, Vendramin et al. [45] assessed the genetic variation among *Abies alba* populations using 2 cpSSRs, while Cubas et al. [46] evaluated the genetic variability and relationships among *Ulex* species using 6 cpSSRs. The detected SSRs were mainly located in the LSC region (67.1%–77%), which is consistent with the characteristics found in other angiosperms [36, 47]. The most abundant type was mononucleotides A/T*,* which is consistent with the results obtained in previous studies [48, 49]. As the plastomes of seven *Saussurea* were conservative, SSR primers can be transferable across species and genera. Therefore, information involving SSRs in this study could be useful for studies at the population level and provide complementary data to the SSR markers of *Saussurea* identified from the nuclear genome [50].

If Ka/Ks is greater than 1, it could indicate that it is under positive selection [26]. Approximately 90% of protein coding genes were below 1 in Ka/Ks values. These results indicate that the protein-coding genes may have undergone purifying selection pressure during their evolution, and it is consistent with the typical tendency shown in the plastid genes [51].

Codon usage bias can also improve our understanding of the effects of natural selection during the evolution [52, 53]. If selective pressure or mutation preferences are absent, synonymous codons prefer equally, and the nucleotide mutations at each amino acid site occur randomly [53]. The tendency for codon preference was similar among species, indicating relatively conserved characteristics of plastome. There were more codons with the RSCU value less than one ended with base C or G and there was high A/T preference in the third codon. These are a common phenomenon in plastomes of vascular plants.

### Phylogenetic relationships

Although Korean endemic *Saussurea* species can be distinguished by morphological characteristics, the low resolution of the previous phylogenetic study (Yun and Kim, unpublished data) was insufficient to understand the relationships between species. In the phylogenetic tree using complete plastomes, Korean endemics were not monophyletic. It is plausible that few independent lineages of *Saussurea* involved in the origin of several endemic species in Korea. In addition, we found that the morphologically similar *S. albifolia* and *S. seoulensis* did not form a sister relationship; *S. albifolia* formed a sister with the group including *S. odontolepis, S. bullockii, S. tianshuiensis,* and *S. chabyoungsanica*. This may suggest that the morphological similarities between *S. albifolia* and *S. seoulensis* could be due to convergent evolution or parallelism. Nevertheless, when the current Korean phylogenetic framework was compared to the previous broader phylogenomic study [16], our result also showed the same relationships between *S. polylepis* and *S. amurensis,* and *S. chabyoungsanica* and *S. tianshuiensis.* However, the clade including the Korean endemics has low support values (bootstrap support value < 50). Although the complete plastome sequences were utilized to build baseline phylogenetic framework among the Korean endemics, the recent explosive speciation of this group perhaps contributed to insufficient resolutions in species relationships.

As for the molecular age estimation based on much broader phylogenomic study [16], the MRCA (Most recent common ancestor) of *S. polylepis* and *S. amurensis* was estimated to be 4.8 MYA, while the MRCA of *S. chabyoungsanica* and *S. tianshuiensis* was 0.32 MYA. Therefore, these age estimates are concordant with the current study. Like precise phylogenetic relationships among species of *Saussurea* in East Asia require further study, accurate molecular age estimation of Korean endemic lineages is needed. In addition, it is yet to be determined whether climatic oscillations during the Pleistocene were major evolutionary drivers in speciation of *Saussurea* [55–57].

## Conclusions

In this study, the complete plastomes of five Korean endemic *Saussurea* were reported and comparatively analyzed these data including previously reported species, *S. chabyoungsanica* and *S. polylepis*. The structures of the plastomes were generally conserved, sharing most genomic features despite the different morphological diversity, but we found the mitochondrial DNA sequences insertion in *S. diamantica*. Through the comparative analyses including variable regions, SSRs, Ka/Ks value, and codon usage, we identified the species-specific SSRs, different patterns of Ka/Ks among species, and nine hotspot regions. These resources will provide insight into the evolution of Korean *Saussurea* and plastome molecular markers for the identification among *Saussurea* species. The phylogenetic tree indicated Korean endemic species did not originate from one lineage.

## Materials and Methods

### Plant materials and DNA extraction

Fresh leaves of *Saussurea albifolia, S. calcicola, S. dimantica, S. grandicapitula,* and *S. seoulensis* were sampled from natural populations in South Korea and dried with silica gel. All voucher specimens were deposited in the Ha Eun Herbarium, at Sungkyunkwan University (SKK) (Table S5). Total genomic DNA was extracted using the DNeasy Plant Mini Kit (Qiagen, Carlsbad, California, USA) according to the manufacturer’s instructions.

### DNA Sequencing, genome assembly, and annotation

After conducting quality control, qualified samples proceeded to library construction. Paired-end libraries were prepared using the TruSeq DNA library preparation kit (Illumina, San Diego, California, USA) according to the standard protocol provided by the manufacturer. DNA sequencing was performed using the Illumina Hiseq 4000 (Illumina, San Diego, California, USA) by Macrogen Corporation (Seoul, Korea). For each species, approximately 3.0 GB raw data were generated. The raw reads were assembled de novo into whole plastomes using Velvet v. 1.2.10 with multiple k-mers [58]. Dual Organellar GenoMe Annotator (DOGMA) software [59] and tRNAscan-SE [60] were used to annotate the protein coding genes and transfer RNA genes, respectively. The graphical maps of plastomes were drawn in the Organellar Genome DRAW (OGDRAW) program [61]. The five annotated complete plastome sequences were submitted to GenBank (Table S5).

### Comparison of plastid genomes

The complete plastomes of the five new species and two previously reported Korean endemic *S. chabyoungsanica* (NC036677) and *S. polylepis* (NC036490) were aligned and adjusted manually using Geneious v.10.2.2. (Biomatters Ltd., Auckland, New Zealand). A large insertion (20,550 bp) found in *S. diamantica* was excluded for analysis. The complete plastomes of the seven Korean endemic *Saussurea* were compared using mVISTA [62] with Shuffle-LAGAN mode [63] and default parameters. The plastome of *S. involucrata* (NC029465) was used as a reference. Sliding window analysis was carried out to calculate the nucleotide diversity (*Pi*) using DnaSP v. 6 [64]. The step size was set to 300 bp, with a 900 bp window length. SSRs were detected using MISA [65]. The minimum repeat thresholds were set to ten for mononucleotide repeats, four for dinucleotide to tetranucleotide repeats, and three for pentanucleotide and hexanucleotide repeats. Sequences of 80 protein-coding regions without stop-codons were extracted from the plastomes of seven *Saussurea* and *S. involucrata* as a reference. KaKs_Calculator 2.0 [66] was used for calculating Ka/Ks values with genetic code 11 (bacterial and plant plastid code) and GY as a calculation mode. The codon usage frequencies and RSCU values for 80 protein-coding genes were determined with DnaSP v.6. The RSCU was divided into four models, including lack of preference (RSCU ≤ 1.0), low preference (1.0 < RSCU< 1.3), moderate preference (1.30 ≤ RSCU ≤ 1.50), and high preference (RSCU > 1.5) [54].

### Phylogenetic analysis and estimation of divergence times

For the phylogenetic analysis, the plastomes of 28 *Saussurea* species including seven Korean endemics and four representative species from four genera (*Arctium, Carthamus, Centaurea,* and *Hemistepta*) as an outgroup were used. Twenty-one additional *Saussurea* species were selected based on the previous study [16]. Sequences were aligned using MAFFT v.7.149 [67] and removed the gap or poorly aligned position using Gblocks v.0.91b, using default settings [68]. A maximum likelihood (ML) analysis was performed in IQ-TREE v. 1.4.2 [69] with 1000 replicates. By using jModelTest v.2.1.10 [70], GTR+I+G was selected as the optimal model based on the Bayesian information criterion. For the bayesian inference (BI) phylogenetic tree, the analysis was performed until the standard deviation of split frequencies was below 0.01 using MrBayes v3.1.2 [71]. Each chain was sampled every 100 generations. The first 25% of the sample was discarded as burn-in, and the rest was used to construct a consensus tree.

Divergence times of Korean endemic *Saussurea* species were estimated from the same dataset used for phylogenetic analysis using BEAST v2.6.2 [72] under lognormal relaxed clock. The GTR model was chosen to generate the tree. As a calibration point, the pairwise divergence time of 11.8 MYA for *Carthamus* and *Centaurea* was applied according to the data deposited in TIMETREE [73]. The Markov chain Monte Carlo chains were set to run for 16 million generations, sampling one every 2,500 generations. Tracer v.1.7 [74] was used for checking the convergence of the chains through adequate effective sample sizes (ESS). Finally, maximum clade credibility trees were calculated in TreeAnnotator v1.8.4 [75] and the summary trees with 95% highest posterior density (HPD) intervals of divergence time were visualized using Figtree v1.4.

## Supplementary Information

**Additional file 1: Figure S1.** Comparison of border regions among the plastomes of seven *Saussurea* species.

**Additional file 2: Figure S2**. Visualization alignment of seven Korean *Saussurea* chloroplast genomes using *S. involucrata* as a reference. The *x*-axis and *y*-scale respectively indicate the base sequence of the alignment and the percentage identity with 50–100%.

**Additional file 3: Figure S3.** The Ka/Ks values of 80 protein-coding genes from seven Korean *Saussurea* plastomes.

**Additional file 4: Table S1.** List of genes found in chloroplast genomes of seven Korean endemic *Saussurea* species. a: IR duplicated gene. b: gene with intron. * *S. diamantica* additionally has mitochondrial *trn*C-GCA and *trn*M-CAU in SSC.

**Additional file 5: Table S2.** The polymorphic regions and single nucleotide polymorphisms shown in group I (*S. calcicola, S. grandicapitula, S. polylepis,* and *S. seoulensis*) and group II (*S. albifolia, S. chabyoungsanica,* and *S. diamantica*).

**Additional file 6: Table S3.** Motif types and numbers of SSRs shown in 15 genes.

**Additional file 7: Table S4.** Codon content of 20 amino acid and stop codons in 80 protein coding genes of the seven cp genomes.

**Additional file 8: Table S5.** List of the five *Saussurea* species newly sequenced in this study. Specimens and assembled sequences are deposited in the Ha Eun Herbarium (Sungkyunkwan University, SKK) and GenBank, respectively.

## Data Availability

Sequence data that support the findings of this study can be downloaded from GenBank (https://www.ncbi.nlm.nih.gov) with the accession codes of MT478053, MN509431, MT536932, MN530094, and MN530095.

## Abbreviations

BI: Bayesian inference
cpDNA: Chloroplast DNA
DnaSP: DNA sequence polymorphism
ESS: Effective sample size
HPD: Highest posterior density
IR: Inverted repeat
Ka: Non–synonymous substitution rate
Ks: Synonymous substitution rate
LSC: Large single copy
ML: Maximum likelihood
MRCA: Most recent common ancestor
MYA: Million years ago
Pi: Nucleotide diversity
Plastome: Plastid genome
RSCU: Relative synonymous codon usage
SNP: Single nucleotide polymorphism
SSC: Small single copy
SSR: Simple sequence repeat

## References

[1] Lipschitz S (1979). Genus *Saussurea* DC.

[2] Butola JS, Samant SS (2010). *Saussurea* species in Indian Himalayan Region: diversity, distribution and indigenous uses. Int J Plant Biol..

[3] Von Raab-Straube E (2003). Phylogenetic relationships in *Saussurea* (Compositae, Cardueae) sensu lato, inferred from morphological, ITS and *trn*L-*trn*F sequence data, with a synopsis of *Himalaiella* gen. nov., *Lipschitziella* and *Frolovia*. Willdenowia..

[4] Kita Y, Fujikawa K, Ito M, Ohba H, Kato M (2004). Molecular phylogenetic analyses and systematics of the genus *Saussurea* and related genera (Asteraceae, Cardueae). Taxon..

[5] Wang YJ, Liu JQ (2004). Phylogenetic analyses of *Saussurea* sect. *Pseudoeriocoryne* (Asteraceae: Cardueae) based on chloroplast DNA *trn*L–F sequences. Biochem Syst Ecol..

[6] Shi Z, Von Raab-Straube E, Wu ZY, Raven PH, Hong DY (2011). Cardueae. Flora of China. Beijing and St.

[7] Wang YJ, Susanna A, Von Raab-Straube E, Milne R, Liu JQ (2009). Island-like radiation of *Saussurea* (Asteraceae: Cardueae) triggered by uplifts of the Qinghai–Tibetan Plateau. Biol J Linn Soc..

[8] Wen J, Zhang J, Nie ZL, Zhong Y, Sun H (2014). Evolutionary diversifications of plants on the Qinghai-Tibetan Plateau. Front Genet..

[9] Palmer JD, Jansen RK, Michaels HJ, Chase MW, Manhart JR (1988). Chloroplast DNA variation and plant phylogeny. Ann Missouri Bot Gard..

[10] Shaw J, Lickey EB, Beck JT, Farmer SB, Liu W, Miller J, Siripun KC, Winder CT, Schilling EE, Small RL (2005). The tortoise and the hare II: Relative utility of 21 noncoding chloroplast DNA sequences for phylogenetic analysis. Am J Bot..

[11] Shaw J, Lickey EB, Schilling EE, Small RL (2007). Comparison of whole chloroplast genome sequences to choose noncoding regions for phylogenetic studies in angiosperms: The tortoise and the hare III. Am J Bot..

[12] Zhang SD, Jin JJ, Chen SY, Chase MW, Soltis DE, Li HT, Yang JB, Li DZ, Yi TS (2017). Diversification of Rosaceae since the Late Cretaceous based on plastid phylogenomics. New Phytol..

[13] Gitzendanner MA, Soltis PS, Wong GKS, Ruhfel BR, Soltis DE (2018). Plastid phylogenomic analysis of green plants: a billion years of evolutionary history. Am J Bot..

[14] Chen HF (2020). Chloroplast Phylogenomics Reveals the Intercontinental Biogeographic History of the Liquorice Genus (Leguminosae: *Glycyrrhiza*). Front Plant Sci..

[15] Zhang X, Deng T, Moore MJ, Ji Y, Lin N, Zhang H, Meng A, Wang H, Sun Y, Sun H (2019). Plastome phylogenomics of *Saussurea* (Asteraceae: Cardueae). BMC Plant Biol..

[16] Xu LS, Herrando-Moraira S, Susanna A, Galbany-Casals M, Chen YS (2019). Phylogeny, origin and dispersal of *Saussurea* (Asteraceae) based on chloroplast genome data. Mol Phylogenet Evol..

[17] Im HT, Saussurea DC, Park CW (2007). The genera of vascular plants of Korea.

[18] Cheon KS, Kim HJ, Han JS, Kim KA, Yoo KO (2017). The complete chloroplast genome sequence of *Saussurea chabyoungsanica* (Asteraceae), an endemic to Korea. Conserv Genet Resour..

[19] Yun SA, Gil HY, Kim SC (2017). The complete chloroplast genome sequence of *Saussurea polylepis* (Asteraceae), a vulnerable endemic species of Korea. Mitochondrial DNA Part B Resour..

[20] Sun EM, Yun SA, Kim SC, Chung GY, Nam MJ, Im HT (2021). *Saussurea albifolia* MJ Nam & HT Im (Compositae), a new species from the Baekdudaegan Area, Korea. J Species Res..

[21] Lee WT, Im HT, Saussurea grandicapitula W. (2007). Lee et HT Im (Compositae), a new species from the Taebaek mountains, Korea. Korean J Pl Taxon.

[22] Im HT, Hong HH, Choi CI. *Saussurea chabyoungsanica* Im (Compositae), a new species from Mt. Chabyoung-san, Korea. J Plant Biol. 1997;40(4):288–290. https://doi.org/10.1007/BF03030462.

[23] Iorizzo M, Senalik D, Szklarczyk M, Grzebelus D, Spooner D, Simon P (2012). De novo assembly of the carrot mitochondrial genome using next generation sequencing of whole genomic DNA provides first evidence of DNA transfer into an angiosperm plastid genome. BMC Plant Biol..

[24] Straub SCK, Cronn RC, Edwards C, Fishbein M, Liston A (2013). Horizontal transfer of DNA from the mitochondrial to the plastid genome and its subsequent evolution in milkweeds (Apocynaceae). Genome Biol Evol..

[25] Ma PF, Zhang YX, Guo ZH, Li DZ (2015). Evidence for horizontal transfer of mitochondrial DNA to the plastid genome in a bamboo genus. Sci Rep..

[26] Yang Z, Bielawski JP (2000). Statistical methods for detecting molecular adaptation. Trends Ecol Evol..

[27] Daniell H, Lin CS, Yu M, Chang WJ (2016). Chloroplast genomes: diversity, evolution, and applications in genetic engineering. Genome Biol..

[28] Goulding SE, Olmstead RG, Morden CW, Wolfe KH (1996). Ebb and flow of the chloroplast inverted repeat. Mol Gen Genet..

[29] Hansen DR, Dastidar SG, Cai Z, Penaflor C, Kuehl JV, Boore JL, Jansen RK (2007). Phylogenetic and evolutionary implications of complete chloroplast genome sequences of four early-diverging angiosperms: *Buxus* (Buxaceae), *Chloranthus* (Chloranthaceae), *Dioscorea* (Dioscoreaceae), and *Illicium* (Schisandraceae). Mol Phylogenet Evol..

[30] Cosner ME, Jansen RK, Palmer JD, Downie SR (1997). The highly rearranged chloroplast genome of *Trachelium caeruleum* (Campanulaceae): multiple inversions, inverted repeat expansion and contraction, transposition, insertions/deletions, and several repeat families. Curr Genet..

[31] Plunkett GM, Downie SR (2000). Expansion and contraction of the chloroplast inverted repeat in Apiaceae subfamily Apioideae. Syst Bot..

[32] Martin W, Stoebe B, Goremykin V, Hansmann S, Hasegawa M, Kowallik KV (1998). Gene transfer to the nucleus and the evolution of chloroplasts. Nature..

[33] Adams KL, Palmer JD (2003). Evolution of mitochondrial gene content: gene loss and transfer to the nucleus. Mol Phylogenet Evol..

[34] Wang D, Wu YW, Shih ACC, Wu CS, Wang YN, Chaw SM (2007). Transfer of chloroplast genomic DNA to mitochondrial genome occurred at least 300 MYA. Mol Biol Evol..

[35] Park I, Yang S, Kim WJ, Song JH, Lee HS, Lee HO, Lee JH, Ahn SN, Moon BC (2019). Sequencing and comparative analysis of the chloroplast genome of *Angelica* polymorpha and the development of a novel indel marker for species identification. Molecules..

[36] Shi H, Yang M, Mo C, Xie W, Liu C, Wu B, et al. Complete chloroplast genomes of two *Siraitia* Merrill species: Comparative analysis, positive selection and novel molecular marker development. PloS One. 2019;14(12):e0226865. 2019. 10.1371/journal.pone.0226865. 10.1371/journal.pone.0226865 PMC6924677 31860647

[37] Cho MS, Yang JY, Yang TJ, Kim SC (2019). Evolutionary Comparison of the Chloroplast Genome in the Woody *Sonchus* Alliance (Asteraceae) on the Canary Islands. Genes..

[38] Li X, Tan W, Sun J, Du J, Zheng C, Tian X, Zeng M, Xiang B, Wang Y (2019). Comparison of Four Complete Chloroplast Genomes of Medicinal and Ornamental *Meconopsis* Species: Genome Organization and Species Discrimination. Sci Rep..

[39] López-Vinyallonga S, Mehregan I, Garcia-Jacas N, Tscherneva O, Susanna A, Kadereit JW (2009). Phylogeny and evolution of the *Arctium-Cousinia* complex (Compositae, Cardueae-Carduinae). Taxon..

[40] Demaio PH, Barfuss MHJ, Kiesling R, Till W, Chiapella JO (2011). Molecular phylogeny of *Gymnocalycium* (Cactaceae): Assessment of alternative infrageneric systems, a new subgenus, and trends in the evolution of the genus. Am J Bot..

[41] Javadi F, Tun YT, Kawase M, Guan K, Yamaguchi H (2011). Molecular phylogeny of the subgenus *Ceratotropis* (genus *Vigna*, Leguminosae) reveals three eco-geographical groups and Late Pliocene-Pleistocene diversification: evidence from four plastid DNA region sequences. Ann Bot..

[42] Michelangeli FA, Guimaraes PJF, Penneys DS, Almeda F, Kriebel R (2013). Phylogenetic relationships and distribution of new world Melastomeae (Melastomataceae). Bot J Linn Soc..

[43] Yazbek M, Oh SH (2013). Peaches and almonds: phylogeny of *Prunus* subg. *Amygdalus* (Rosaceae) based on DNA sequences and morphology. Plant Syst Evol..

[44] Powell W, Machray GC, Provan J (1996). Polymorphism revealed by simple sequence repeats. Trends Plant Sci..

[45] Vendramin GG, Degen B, Petit RJ, Anzidei M, Madaghiele A, Ziegenhagen B (1999). High level of variation at *Abies alba* chloroplast microsatellite loci in Europe. Mol Ecol..

[46] Cubas P, Pardo C, Tahiri H (2005). Genetic variation and relationships among *Ulex* (Fabaceae) species in southern Spain and northern Morocco assessed by chloroplast microsatellite (cpSSR) markers. Am J Bot..

[47] Wei F, Tang D, Wei K, Qin F, Li L, Lin Y, Zhu Y, Khan A, Kashif MH, Miao J (2020). The complete chloroplast genome sequence of the medicinal plant *Sophora tonkinensis*. Sci Rep..

[48] Cui Y, Zhou J, Chen X, Xu Z, Wang Y, Sun W, Song J, Yao H (2019). Complete chloroplast genome and herbaria comparative analysis of three *Lycium* (Solanaceae) species with medicinal and edible properties. Gene Rep..

[49] Gao K, Li J, Khan WU, Zhao T, Yang X, Yang X, Guo B, An X (2019). Comparative genomic and phylogenetic analyses of *Populus* section *Leuce* using complete chloroplast genome sequences. Tree Genet Genomes..

[50] Yun SA, Kim SC (2019). Microsatellite markers for *Saussurea polylepis* (Asteraceae), a vulnerable continental island species endemic to Korea. Appl Plant Sci..

[51] Guisinger MM, Kuehl JV, Boore JL, Jansen RK (2008). Genome-wide analyses of Geraniaceae plastid DNA reveal unprecedented patterns of increased nucleotide substitutions. Proc Natl Acad Sci U.S.A..

[52] Xu C, Dong J, Tong C, Gong X, Wen Q, Zhuge Q (2013). Analysis of synonymous codon usage patterns in seven different *Citrus* species. Evol Bioinform..

[53] Ingvarsson PK (2008). Molecular evolution of synonymous codon usage in *Populus*. BMC Evol Biol..

[54] Yu X, Zuo L, Lu D, Lu B, Yang M, Wang J (2019). Comparative analysis of chloroplast genomes of five *Robinia* species: Genome comparative and evolution analysis. Gene..

[55] Hewitt GM (1996). Some genetic consequences of ice ages and their role in divergence and speciation. Biol J Linn Soc..

[56] Hewitt GM (2000). The genetic legacy of the Quaternary ice ages. Nature..

[57] Schmitt T (2007). Molecular biogeography of Europe: Pleistocene cycles and postglacial trends. Front Zool..

[58] Zerbino DR, Birney E (2008). Velvet: Algorithms for de novo short read assembly using de Bruijn graphs. Genome Res..

[59] Wyman SK, Jansen RK, Boore JL (2004). Automatic annotation of organellar genomes with DOGMA. Bioinformatics..

[60] Lowe TM, Chan PP (2016). tRNAscan-SE on-line: Integrating search and context for analysis of transfer RNA genes. Nucleic Acids Res..

[61] Lohse M, Drechsel O, Bock R (2007). Organellar genome DRAW(OGDRAW): A tool for the easy generation of high-quality custom graphical maps of plastid and mitochondrial genomes. Curr Genet..

[62] Frazer KA, Pachter L, Poliakov A, Rubin EM, Dubchak I (2014). VISTA: Computational tools for comparative genomics. Nucleic Acids Res..

[63] Brudno M, Malde S, Poliakov A, Do CB, Couronne O, Dubchak I, Batzoglou S (2003). Glocal alignment: Finding rearrangements during alignment. Bioinformatics..

[64] Rozas J, Ferrer-Mata A, Sánchez-DelBarrio JC, Guirao-Rico S, Librado P, Ramos-Onsins SE, Sánchez-Gracia A (2017). DnaSP v6: DNA sequence polymorphism analysis of large datasets. Mol Biol Evol..

[65] Thiel T, Michalek W, Varshney RK, Graner A (2003). Exploiting EST databases for the development and characterization of gene-derived SSR-markers in barley (*Hordeum vulgare* L.). Theor Appl Genet..

[66] Wang D, Zhang Y, Zhang Z, Zhu J, Yu J (2010). KaKs_Calculator 2.0: a toolkit incorporating gamma-series methods and sliding window strategies. Genomics Proteomics Bioinformatics..

[67] Katoh K, Standley DM (2013). MAFFT multiple sequence alignment software version 7: improvements in performance and usability. Mol Biol Evol..

[68] Castresana J (2000). Selection of conserved blocks from multiple alignments for their use in phylogenetic analysis. Mol Biol Evol..

[69] Nguyen LT, Schmidt HA, Von Haeseler A, Minh BQ (2015). IQ-TREE: A fast and effective stochastic algorithm for estimating maximum-likelihood phylogenies. Mol Biol Evol..

[70] Darriba D, Taboada GL, Doallo R, Posada D (2012). jModelTest 2: more models, new heuristics and parallel computing. Nat Methods..

[71] Ronquist F, Huelsenbeck JP (2003). MrBayes 3: Bayesian phylogenetic inference under mixed models. Bioinformatics..

[72] Bouckaert R, Heled J, Kühnert D, Vaughan T, Wu CH, Xie D, Suchard MA, Rambaut A, Drummond AJ (2004). BEAST 2: A software platform for Bayesian evolutionary analysis. PLoS Comput Biol..

[73] Hedges SB, Dudley J, Kumar S (2006). TimeTree: a public knowledge-base of divergence times among organisms. Bioinformatics..

[74] Rambaut A, Drummond AJ, Xie D, Baele G, Suchard MA (2018). Posterior summarization in Bayesian phylogenetics using Tracer 1.7. Syst Biol..

[75] Drummond A, Suchard MA, Xie D, Rambaut A (2012). Bayesian Phylogenetics with Beauti and the Beast 1.7. Mol Biol Evol..

