# Supplementary material for: Comparative plastomes and phylogenetic analysis of seven Korean endemic Saussurea (Asteraceae)
Source: BMC Plant Biol. 2022 Nov 29;22:550. doi: 10.1186/s12870-022-03946-6 (PMC9706989; doi:10.1186/s12870-022-03946-6)
Supplement: Supplementary file 1 — Additional file 1: Figure S1. Comparison of border regions among the plastomes of seven Saussurea species. [file 12870_2022_3946_MOESM1_ESM.docx]

**Table S2** The polymorphic regions and single nucleotide polymorphisms shown in group I (*S. calcicola, S. grandicapitula, S. polylepis,* and *S. seoulensis*) and group II (*S*. *albifolia, S. chabyoungsanica,*and *S. diamantica*)

| **Region** | | **I group** | **II group** | **Region** | | **I group** | **II group** | **Region** | | **I group** | **II group** |
| --- | --- | --- | --- | --- | --- | --- | --- | --- | --- | --- | --- |
| *mat*K | LSC | A | G | *ycf*3-*trn*S | LSC | C | A | *psb*B | LSC | C | A |
| *rps*16 intron | LSC | T | A | *trn*S-*rps*4 | LSC | T | C | *pet*B intron | LSC | G | T |
| *rps*16-*trn*Q | LSC | T | C | *ndh*C-*trn*V | LSC | A | G | *pet*B intron | LSC | G | T |
| *trn*Q-*psb*K | LSC | A | T | *ndh*C-*trn*V | LSC | T | C | *pet*D intron | LSC | T | C |
| *trn*S-*trn*C | LSC | A | G | *atp*B | LSC | A | G | *rps*11-*rpl*36 | LSC | A | G |
| *trn*C-*pet*N | LSC | T | G | *rbc*L | LSC | A | C | *rpl*14 | LSC | G | A |
| *trn*D-*trn*Y | LSC | G | A | *acc*D | LSC | T | C | *rps*19-*rpl*2 | IR | T | G |
| *trn*E-*rpo*B | LSC | C | A | *acc*D | LSC | T | C | *ycf*1 | SSC | G | T |
| *rpo*B | LSC | A | G | *acc*D | LSC | A | G | *ycf*1 | SSC | T | A |
| *rpo*C2 | LSC | G | T | *acc*D | LSC | T | C | *ycf*1 | SSC | G | T |
| *rpo*C2 | LSC | C | A | *acc*D-*psa*I | LSC | G | T | *rps*15 | SSC | A | C |
| *rpo*C2 | LSC | A | G | *acc*D-*psa*I | LSC | T | G | *ndh*A intron | SSC | G | A |
| *atp*I | LSC | A | G | *acc*D-*psa*I | LSC | G | T | *ndh*A intron | SSC | T | C |
| *atp*I-*atp*H | LSC | A | G | *ycf*4-*cem*A | LSC | G | C | *psa*C-*ndh*D | SSC | A | T |
| *atp*I-*atp*H | LSC | T | C | *ycf*4-*cem*A | LSC | C | G | *ndh*D | SSC | A | G |
| *trn*T-*psb*D | LSC | C | T | *psb*E-*pet*L | LSC | G | T | *ndh*D | SSC | A | C |
| *trn*T-*psb*D | LSC | T | G | *psb*E-*pet*L | LSC | A | T | *ndh*D-*ccs*A | SSC | G | T |
| *trn*fM-*rps*14 | LSC | A | G | *pet*G-*trn*W | LSC | C | A | *rpl*32-*ndh*F | SSC | C | G |
| *rps*14 | LSC | A | C | *psa*J-*rpl*33 | LSC | A | C | *rpl*32-*ndh*F | SSC | A | G |
