# Supplementary material for: Comparative plastomes and phylogenetic analysis of seven Korean endemic Saussurea (Asteraceae)
Source: BMC Plant Biol. 2022 Nov 29;22:550. doi: 10.1186/s12870-022-03946-6 (PMC9706989; doi:10.1186/s12870-022-03946-6)
Supplement: Supplementary file 3 — Additional file 3: Figure S3. The Ka/Ks values of 80 protein-coding genes from seven Korean Saussurea plastomes. [file 12870_2022_3946_MOESM3_ESM.docx]

**Table S5.** List of the five *Saussurea* species newly sequenced in this study. Specimens and assembled sequences are deposited in the Ha Eun Herbarium (Sungkyunkwan University, SKK) and GenBank, respectively.

| **Taxon** | **Specimen Number** | **GenBank Accession** |
| --- | --- | --- |
| *S. albifolia* | SKK130926019 | MT478053 |
| *S. calcicola* | SKK170929006 | MN509431 |
| *S. diamantica* | SKK171015001 | MT536932 |
| *S. grandicapitula* | SKK131004040 | MN530094 |
| *S. seoulensis* | SKK171022002 | MN530095 |
