# Supplementary figures and images for: Comparative plastomes and phylogenetic analysis of seven Korean endemic Saussurea (Asteraceae)
Source: BMC Plant Biol. 2022 Nov 29;22:550. doi: 10.1186/s12870-022-03946-6 (PMC9706989; doi:10.1186/s12870-022-03946-6)

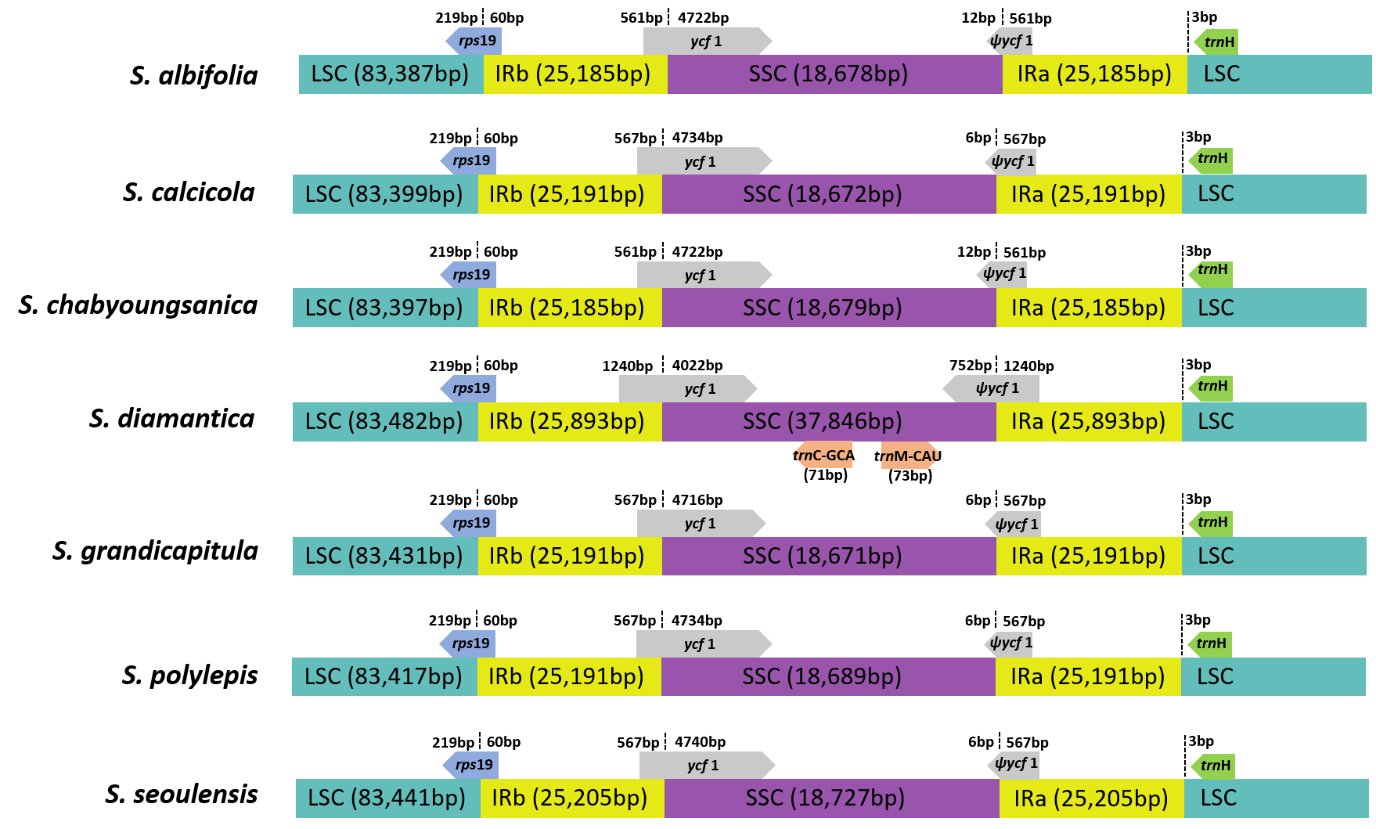


**Figure S1** Comparison of border regions among the plastomes of seven *Saussurea* species

Supplement: Supplementary file 4 — Additional file 4: Table S1. List of genes found in chloroplast genomes of seven Korean endemic Saussurea species. a: IR duplicated gene. b: gene with intron. * S. diamantica additionally has mitochondrial trnC-GCA and trnM-CAU in SSC. [file 12870_2022_3946_MOESM4_ESM.docx]

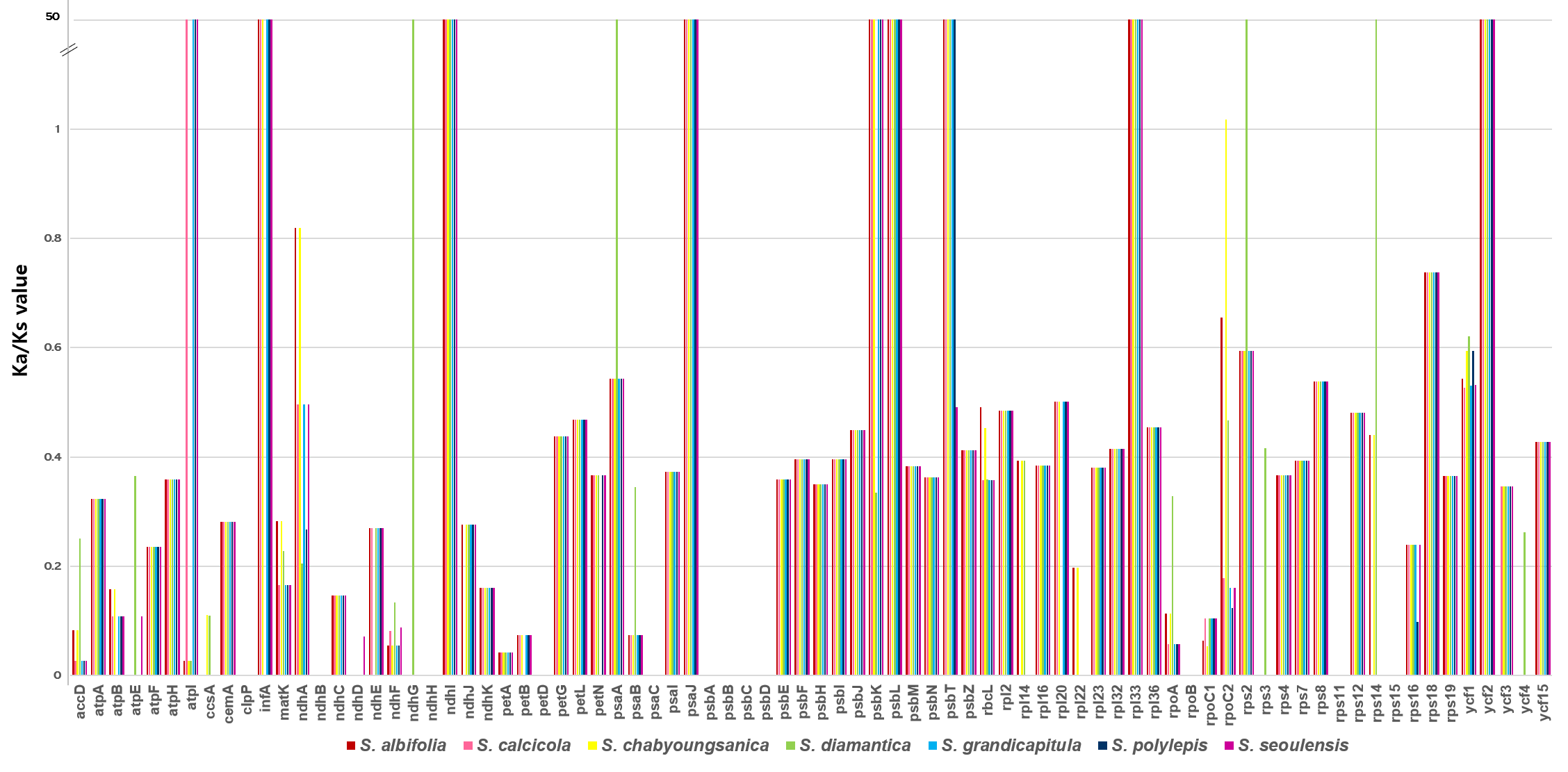


**Figure S3** The Ka/Ks values of 80 protein-coding genes from seven Korean *Saussurea* plastomes

Supplement: Supplementary file 7 — Additional file 7: Table S4. Codon content of 20 amino acid and stop codons in 80 protein coding genes of the seven cp genomes. [file 12870_2022_3946_MOESM7_ESM.docx]
