# Supplementary material for: Comparative plastomes and phylogenetic analysis of seven Korean endemic Saussurea (Asteraceae)
Source: BMC Plant Biol. 2022 Nov 29;22:550. doi: 10.1186/s12870-022-03946-6 (PMC9706989; doi:10.1186/s12870-022-03946-6)
Supplement: Supplementary file 5 — Additional file 5: Table S2. The polymorphic regions and single nucleotide polymorphisms shown in group I (S. calcicola, S. grandicapitula, S. polylepis, and S. seoulensis) and group II (S. albifolia, S. chabyoungsanica, and S. diamantica). [file 12870_2022_3946_MOESM5_ESM.docx]

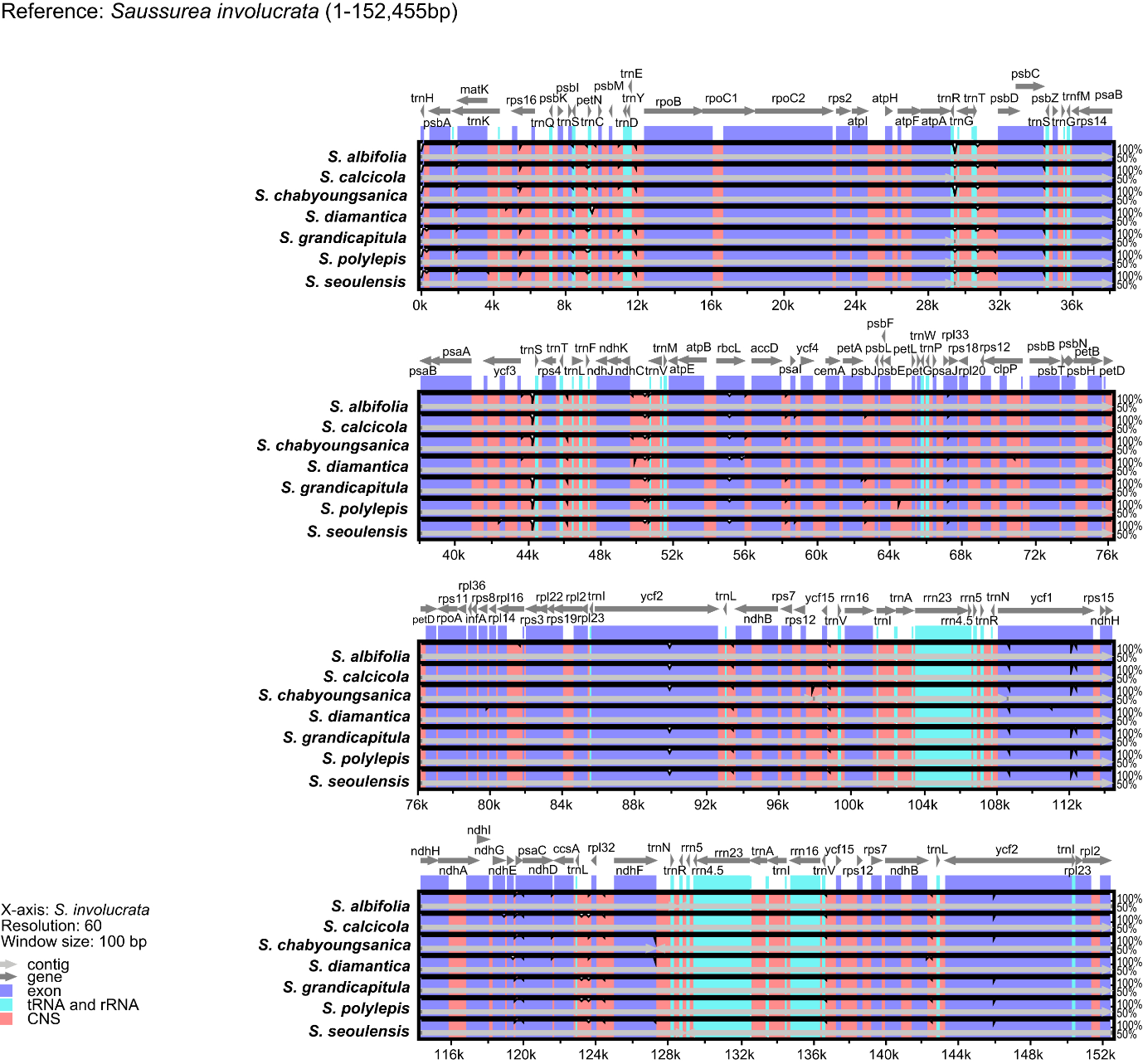


**Figure S2** Visualization alignment of seven Korean *Saussurea* chloroplast genomes using *S. involucrata* as a reference. The *x*-axis and *y*-scale respectively indicate the base sequence of the alignment and the percentage identity with 50–100%
