# Supplementary material for: Comparative plastomes and phylogenetic analysis of seven Korean endemic Saussurea (Asteraceae)
Source: BMC Plant Biol. 2022 Nov 29;22:550. doi: 10.1186/s12870-022-03946-6 (PMC9706989; doi:10.1186/s12870-022-03946-6)
Supplement: Supplementary file 6 — Additional file 6: Table S3. Motif types and numbers of SSRs shown in 15 genes. [file 12870_2022_3946_MOESM6_ESM.docx]

**Table S3** Motif types and numbers of SSRs shown in 15 genes

| region | motif | ***S. albifolia*** | ***S.calcicola*** | ***S.chabyoungsanica*** | ***S.diamantica*** | ***S.grandicapitula*** | ***S.polylepis*** | ***S.seoulensis*** |
| --- | --- | --- | --- | --- | --- | --- | --- | --- |
|  |  | repeat number | | | | | | |
| *cem*A | TC | 4 | 4 | 4 | 4 | 4 | 4 | 4 |
| *ndh*B | AG | 4 | 4 | 4 | 4 | 4 | 4 | 4 |
| *pet*A | AT | 4 | 4 | 4 | 4 | 4 | 4 | 4 |
| *psa*A | AG | 4 | 4 | 4 | 4 | 4 | 4 | 4 |
| *psb*C | TTC | 4 | 4 | 4 | 4 | 4 | 4 | 4 |
| *rbc*L | GA | 4 | 4 | 4 | 4 | 4 | 4 | 4 |
| *rpo*A | TA | 4 | 4 | 4 | 0 | 4 | 4 | 4 |
| *rpo*A | T | 10 | 10 | 10 | 0 | 10 | 10 | 10 |
| *rpo*B | A | 10 | 10 | 10 | 10 | 10 | 10 | 10 |
| *rpo*C1 | A | 10 | 10 | 10 | 10 | 10 | 10 | 10 |
| *rpo*C1 | A | 0 | 0 | 10 | 0 | 0 | 0 | 0 |
| *rpo*C1 | TA | 5 | 5 | 5 | 5 | 5 | 5 | 5 |
| *rpo*C2 | AT | 5 | 5 | 5 | 5 | 5 | 5 | 5 |
| *rpo*C2 | TATTCC | 3 | 3 | 3 | 3 | 3 | 0 | 3 |
| *rps*15 | GA | 4 | 4 | 4 | 4 | 4 | 4 | 4 |
| *rrn*23 | AG | 4 | 4 | 4 | 4 | 4 | 4 | 4 |
| *trn*S-UGA | GA | 4 | 4 | 4 | 4 | 4 | 4 | 4 |
| *ycf*1 | AT | 4 | 4 | 4 | 4 | 4 | 4 | 4 |
| *ycf*1 | TA | 4 | 4 | 4 | 4 | 4 | 4 | 4 |
| *ycf*1 | TACAAA | 0 | 0 | 0 | 0 | 0 | 0 | 3 |
| *ycf*2 | GA | 4 | 4 | 4 | 4 | 4 | 4 | 4 |
| *ycf*2 | GA | 4 | 4 | 4 | 4 | 4 | 4 | 4 |
| *ycf*2 | GA | 4 | 4 | 4 | 4 | 4 | 4 | 4 |
