# Supplementary material for: Comparative plastomes and phylogenetic analysis of seven Korean endemic Saussurea (Asteraceae)
Source: BMC Plant Biol. 2022 Nov 29;22:550. doi: 10.1186/s12870-022-03946-6 (PMC9706989; doi:10.1186/s12870-022-03946-6)
Supplement: Supplementary file 8 — Additional file 8: Table S5. List of the five Saussurea species newly sequenced in this study. Specimens and assembled sequences are deposited in the Ha Eun Herbarium (Sungkyunkwan University, SKK) and GenBank, respectively. [file 12870_2022_3946_MOESM8_ESM.docx]

**Table S1** List of genes found in chloroplast genomes of seven Korean endemic *Saussurea* species

| Category for genes | Group of gene | Name of gene |
| --- | --- | --- |
| Photosynthesis | ATP synthase | *atp*A, *atp*B, *atp*E, *atp*F^b^, *atp*H, *atp*I, *ycf*2^a^ |
|  | NADH-dehydrogenase | *ndh*A^b^, *ndh*B^ab^, *ndh*C, *ndh*D, *ndh*E, *ndh*F, *ndh*G, *ndh*H, *ndh*I, *ndh*J, *ndh*K |
|  | Cytochrome b/f complex | *pet*A, *pet*B^b^, *pet*D^b^, *pet*G, *pet*L, *pet*N |
|  | Cytochrome c synthesis | *ccs*A |
|  | Photosystem I | *psa*A, *psa*B, *psa*C, *psa*I, *psa*J |
|  | Photosystem II | *psb*A, *psb*B, *psb*C, *psb*D, *psb*E, *psb*F, *psb*H, *psb*I, *psb*J, *psb*K, *psb*L, *psb*M, *psb*N, *psb*T, *psb*Z |
|  | Photosystem I assembly | *ycf*3^b^, *ycf*4 |
|  | Rubisco large subunit | *rbc*L |
| Transcription and translation related genes | Ribosomal protein,  large subunit | *rpl*2^ab^, *rpl*14, *rpl*16^b^, *rpl*20, *rpl*22, *rpl*23^a^, *rpl*32, *rpl*33, *rpl*36 |
|  | Ribosomal protein,  small subunit | *rps*2, *rps*3, *rps*4, *rps*7^a^, *rps*8, *rps*11, *rps*12, *rps*14, *rps*15, *rps*16^b^, *rps*18, *rps*19 |
|  | RNA polymerase | *rpo*A, *rpo*B, *rpo*C1^b^, *rpo*C2 |
|  | Translation initiation factor | *inf*A |
| RNA genes | Ribosomal RNA | *rrn*4.5^a^, *rrn*5^a^, *rrn*16^a^, *rrn*23^a^ |
|  | Transfer RNA* | *trn*A-UGC^ab^, *trn*C-GCA, *trn*D-GUC, *trn*E-UUC, *trn*F-GAA, *trn*fM-CAU, *trn*G-GCC, *trn*G-UCC^b^, *trn*H-GUG, *trn*I-CAU^a^, *trn*I-GAU^ab^, *trn*K-UUU^b^, *trn*L-CAA^a^, *trn*L-UAA^b^, *trn*L-UAG, *trn*M-CAU, *trn*N-GUU^a^, *trn*P-UGG, *trn*Q-UUG, *trn*R-ACG^a^, *trn*R-UCU, *trn*S-GCU, *trn*S-GGA, *trn*S-UGA, *trn*T-CGU, *trn*T-UGU, *trn*V-GAC^a^, *trn*V-UAC^b^, *trn*W-CCA, *trn*Y-GUA |
| Other protein-coding genes | Maturase | *mat*K |
|  | Protease | *clp*P^b^ |
|  | Envelope membrane protein | *cem*A |
|  | Subunit Acetyl-CoA-carboxylate | *acc*D |
|  | Translocon | *ycf*1 |
| Unknown | Conserved open reading frames | *ycf*15^a^ |

a: IR duplicated gene

b: gene with intron

* indicates that *S. diamantica* additionally has mitochondrial *trn*C-GCA and *trn*M-CAU in SSC.
